# Clinical-epidemiological evaluation of victims of thoracic trauma in a reference hospital in Aracaju-SE

**DOI:** 10.1590/0100-6991e-20233542-en

**Published:** 2023-10-30

**Authors:** Hélder Santos Gonçalves, Mateus Lenier Rezende, Isadora Valentina dos Santos Cunha, Alan Silva Cesar, Flávio Luiz Dósea Cabral, Leda Maria Delmondes Freitas Trindade

**Affiliations:** 1 - Universidade Tiradentes, Curso de Medicina - Aracaju - SE - Brasil

**Keywords:** Epidemiology, Chest Pain, Multiple Trauma, Thoracic Injuries, Lung Injury, Epidemiologia, Tórax, Traumatismo Múltiplo, Traumatismos Torácicos, Lesão Pulmonar

## Abstract

**Introduction:**

thoracic trauma is defined as anything that involves the rib cage, the musculoskeletal framework that houses the heart, lungs, pleurae and mediastinal structures. It can be superficial or immediately lifethreatening for victims. In Brazil, most assistance is due to urban violence.

**Objective:**

evaluate the clinical and epidemiological aspect of patients who are victims of thoracic trauma treated at Hospital de Urgência de Sergipe, Aracaju/SE, Brazil.

**Method:**

cross-sectional, observational and prospective study, carried out for eleven months, with 100 polytraumatized patients. A semi-structured form was applied, and the data were systematized, analyzed and statistically tested considering a 5% margin of error. Results: 85% of the patients were male, with a mean age of 39.3 and an age range of 30 to 49 years; 57% of them had incomplete primary education, 70% had a family income of up to 2 minimum wages and 41% were from Greater Aracaju. As for the mechanism of trauma, 33% were car-related, with blunt trauma as the main mechanism, and rib fractures as the main consequence. Among penetrating injuries, CWI (26%) and GSW (21%) were the most prevalent, with hemothorax being the main consequence. Most patients underwent thoracostomy (59%).

**Conclusion:**

the profile found was of young men, victims of urban violence. The thoracostomy was resolving in most cases and should be instituted promptly when necessary. A smaller number of patients may require thoracotomy, especially in the presence of hemodynamic instability.

## RESUMO

**Introdução:**

o trauma torácico é definido como toda aquele que envolve a caixa torácica, arcabouço osteomuscular que aloja o coração, os pulmões, as pleuras e as estruturas do mediastino. Pode ser superficial ou causar risco de vida imediato às vítimas. No Brasil, a maioria dos atendimentos é decorrente da violência urbana.

**Objetivo:**

avaliar o aspecto clínico-epidemiológico dos pacientes vítimas de trauma torácico atendidos no Hospital de Urgência de Sergipe, Aracaju/SE, Brasil.

**Métodos:**

estudo transversal, observacional e prospectivo, realizado durante onze meses, com 100 pacientes politraumatizados. Formulário semiestruturado foi aplicado, e os dados sistematizados, analisados e testados estatisticamente considerando-se a margem de erro de 5%. Resultados: 85% dos pacientes eram do gênero masculino, com idade média de 39,3 e faixa etária de 30 a 49 anos; 57% deles possuíam ensino fundamental incompleto, 70% tinham renda familiar de até 2 salários mínimos e 41% eram provenientes da Grande Aracaju. Quanto ao mecanismo de trauma, 33% foi automobilístico, tendo como principal mecanismo o trauma contuso, e a principal consequência a fratura de arcos costais. Dentre os ferimentos penetrantes, os FAB (26%) e FAF (21%) foram os mais prevalentes, sendo o hemotórax sua principal consequência, sendo maioria dos pacientes submetida a toracostomia (59%).

**Conclusão:**

o perfil encontrado foi de homens, jovens, vítimas da violência urbana. A toracostomia foi resolutiva na maioria dos casos e deve ser instituída prontamente quando necessário. Uma menor parte dos pacientes necessitou de toracotomia, principalmente na presença de instabilidade hemodinâmica.

## INTRODUCTION

The term trauma derives from the Greek traumatos, which means wound, damage, or harm, and is used in medical vocabulary to refer to the organic consequences caused by injuries resulting from external violence^1^ ^,^ ^2^. According to the American College of Surgeons Committee on Trauma (ACSCOT), it can also be defined as damage characterized by structural changes or physiological imbalance, resulting from acute exposure to some form of energy, be it mechanical, electrical, thermal, chemical, or radioactive, affecting superficially soft parts and/or damaging noble and deep structures in the body^2^ ^-^ ^4^.

The majority of traumas result from car accidents, runovers, falls, gunshots, stabbing, among others^2^ ^,^ ^3^. With its high prevalence and high association with morbidity and productive losses, both in developed and developing countries, trauma has come to be considered a public health problem, especially in the younger population. In this sense, the WHO estimates that 2.4 million people will die in traffic in 2030^4^ ^,^ ^5^.

In Brazil, trauma is the main cause of death in the group up to 40 years of age, and the third in relation to the general population, involving, in addition to costs that exceed BRL nine billion, important social issues, with high expenses in hospitalization, insurance, labor charges, and reduced productivity. Furthermore, physical assaults, car accidents, and other forms of urban violence represented 12.5% of total deaths and are among the main causes^3^ ^,^ ^6^ ^,^ ^7^.

Thoracic trauma (TT) accounts for 25% of death cases and represents 10% to 15% of total traumas in the world, being characterized by high mortality, mainly due to the involvement of cardiovascular structures^3^ ^,^ ^4^ ^,^ ^6^. In the United States, this is the third most lethal type of trauma, behind only traumatic brain injury and extremity trauma. In Brazil, it is the second most common type (7.3% of occurrences), behind only extremity trauma^6^.

TT can be defined as anything that involves the transmission of energy to the thoracic cage, that is, the external musculoskeletal framework that houses the mediastinal structures and organs, and which is located between the neck and the abdomen8. Depending on each event, thoracic injuries can be divided into four large groups: chest wall, pulmonary, mediastinal, and diaphragmatic^3^ ^,^ ^4^ ^,^ ^6^.

Furthermore, it can be classified into two main types: penetrating (or open) and blunt (or closed). In the first case, it is generally abrupt and results from the direct application of a mechanical force on a small area on the thoracic surface, resulting in discontinuity of the skin, and is usually caused by gunshot (GSW) or stabbing weapons (SW). In the second, the energy resulting from the event is transmitted to the body’s internal structures and there is no loss of skin integrity, such as injuries caused by aggression, collision, fall from higher level, runover and crushing^3^ ^,^ ^4^ ^,^ ^6^. In both cases, the spectrum of the injury will determine its severity. However, blunt traumas are usually more serious than penetrating ones, even though they are relatively less frequent.

For the diagnosis of thoracic injuries, initial suspicion begins with a physical examination, followed by a chest x-ray; however, both have low sensitivity^3^. Regarding imaging exams, radiography has a sensitivity close to 68% and a specificity of 76%, compared to computed tomography (CT), whose sensitivity and specificity are close to 100%. This is the most sensitive method, although it must be used with caution, since its effectiveness does not always compensate for the potential risks related to the time spent to perform it and the higher costs^6^ ^,^ ^9^. On the other hand, Focused Abdominal Ultrasound for Trauma, with the extended protocol for thoracic examination (E-FAST), has been gaining importance to exclude pneumo and hemothorax, being reported as more efficient than radiography^9^ ^,^ ^10^.

Bearing in mind that it is of fundamental importance for any doctor who works in emergency departments to know the mechanisms of trauma, its epidemiology and the main injuries for taking action, the purpose of the present study is to analyze the clinical and epidemiological profile of patients admitted due to chest trauma at the Hospital de Urgência de Sergipe (HUSE), a Brazilian hospital located in the state of Sergipe, encompassing its complications and the treatment indicated for each patient.

## METHODS

### Project and Patients

This is an observational, prospective, and descriptive study, developed from February 2022 to January 2023, carried out at the HUSE, a reference in trauma care in the state of Sergipe, in addition to patients from Bahia, Alagoas, and Pernambuco. To this end, we included 100 victims of chest trauma admitted to the service, with ages between 15 and 90 years. Table 1 describes the inclusion and exclusion criteria.

**Table 1:** Study inclusion and exclusion criteria.

Inclusion criteria	Exclusion Criteria
Patients hospitalized or admitted to the hospital diagnosed with chest injuries resulting from trauma	Patients with any acute or chronic condition that prevents them from agreeing to the terms of the research or answering the questions, and without the presence of a legal guardian or companion
Patients who have already undergone treatment for their injuries	Patients and/or guardian who were unable to provide sufficient information to complete the form
	Patients or companions who refused to sign the ICF and IAF, for those under 18 years of age

ICF: Informed Consent Form. IAF: Informed Assent Form. Source: Authors.

### Instruments and Data Collection

We pre-prepared and used a form to collect data, based on the literature, completed based on bedside interviews and analysis of medical records. The recorded participant’s data contained the following variables: Personal Data: name, date of birth, age, sex, origin, place of birth, profession, level of education; and Clinical Data: type of trauma, injuries present in the chest, injuries present on the body.

### Statistical analysis

We organized the data into tables and figures presented in the form of absolute numbers. To characterize the studied population, we calculated measures of central tendency, such as mean and standard deviation. The data were consolidated in an Excel spreadsheet and analyzed using the R software, version 4.0.0. We used the chi-square test to compare proportions. The significance level used was 0.05.

### Ethical Approval and Consent to Participate

The research was approved by the Ethics in Research Committee of Universidade Tiradentes (CEP-UNIT), CAAE: 28342819.3.0000.5371. Every participant signed the ICF and IAF, for those under 18 years of age.

## RESULTS

We included 100 patients in the study, 85 male (85%) and 15 female (15%). The average age was 38.25 for males (range 17 83) and 45.47 for females (range 15 90). The highest prevalence was among adults between the ages of 30 and 49 (40%), followed by young adults aged 18 to 29 (32%), adults over 50 (25%), and children under 18 (3%). Regarding education, most patients had incomplete junior high (57%), 49 (57.6%) of the patients were male, and only five (5%) had completed university (Table 2).

**Table 2:** Epidemiology of Chest Trauma.

	Distribution by Sex			
	n (%)	Mean Age	Variance	Standard deviation
Male	85 (85%)	38.25	-	± 22.7
Female	15 (15%)	45.47	-	± 14.8
Total	100 (100%)	39.33	270.72	± 16.45
	Distribution by Age Group			
	n (%)	Men (%)	Women (%)	
Under 18	3 (3%)	2 (2.4%)	1 (6.7%)	
18-29	32 (32%)	28 (32.9%)	4 (26.7%)	
30-49	40 (40%)	35 (41.2%)	5 (33.3%)	
Over 50	25 (25%)	20 (23.5%)	5 (33.3%)	
Total	100	85	15	
	Distribution by Education Level			
	n (%)	Men (%)	Women (%)	
No Instruction	7 (7%)	4 (4.7%)	3 (20%)	
Incomplete Junior High	57 (57%)	49 (57.6%)	8 (53.3%)	
	Distribution by Education Level			
	n (%)	Men (%)	Women (%)	
Complete Junior High	13 (13%)	12 (14.1%)	1 (6.7%)	
Incomplete Highschool	8 (8%)	7 (8.2%)	1 (6.7%)	
Complete Highschool	9 (9%)	8 (9.4%)	1 (6.7%)	
Incomplete University	1 (1%)	1 (1.2%)	0 (0%)	
Complete University	5 (5%)	4 (4.7%)	1 (6.7%)	

n: absolute frequency. Source: Authors.

**Figure 1:**
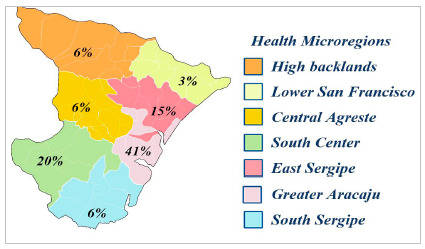
Division of patients treated according to microregions in the State of Sergipe.

As for average family income, 70% have no income or an income of less than two minimum wages, 22% have between two and four, and 8% have four to eight wages (Table 3). Regarding origin, 41% were from Greater Aracaju, which encompasses the capital and adjacent municipalities. The rest came from municipalities in the interior of the State and 3% of patients were from other states (two from Alagoas and one from Bahia). The regions of the state were divided according to the Sergipe health microregions (Figure 1). 

**Table 3:** Distribution by Income

	n (%)	Men	Women
Up to 2 minimum wages	70 (70%)	60	10
From 2 to 4 minimum wages	22 (22%)	18	4
From 4 to 10 minimum wages	8 (8%)	7	1
Above 10 minimum wages	0 (0%)	0	0

n: absolute frequency. *Adjusted minimum wage for 2022: R$1,212.00. Source: Authors.

Regarding the mechanism of trauma, automobile accidents were the cause of most cases, around 33%, followed by SW (26%), GSW (21%), fall from high level (13%), assault (3%), sports injuries (1%), and 3 cases that did not fit into the categories described above, with the presence of a statistically relevant association of fall from high level with the female sex (Table 4).

**Table 4:** Distribution of Epidemiology According to Mechanism.

	n (%)	Assault	Sport	Vehicles	Fall from Level	SW	GSW	Other
Distribution by Sex								
Men	85 (85%)	3	1	28	8	23	20	two
Women	15 (15%)	0	0	5	5	3	1	1
p-Value*	-	0.46004	0.67287	0.97624	0.011089	0.56554	0.13933	0.366555
Age Groups								
Under 18	3 (3%)	0	0	1	1	0	1	0
18-29	32 (32%)	0	0	6	1	11	14	0
30-49	40 (40%)	2	0	15	3	13	6	1
Over 50	25 (25%)	1	1	11	8	2	0	2

n: absolute frequency. GSW: gunshot wounds. SW: stab wounds. *Chi-Square Test. Source: Authors.

Regarding the means of transportation involved in the trauma events, we observed that 67% were caused by motorcycle accidents, 12.4% by car accidents or larger vehicles, 6% by animal-drawn vehicles and bicycles, and 9% runovers (Table 5). 

**Table 5:** Distribution of accidents by means of transportation.

	n (%)	Men	Women	p-value*
Motorcycle	22 (67%)	19	3	0.73137
Cars or other larger vehicles	4 (12%)	4	0	0.367291
Animal drawn vehicle	2 (6%)	2	0	0.537505
Bicycle	2 (6%)	2	0	0.537505
Runover	3 (9%)	2	1	0.356957

n: absolute frequency. *Chi-Square Test. Source: Authors.

In motorcycle accidents, 5% occurred in people under 18 years of age, 14% in young people aged 18 to 29 years, 45% in the age group between 30 and 49 years, and 36% in patients over 50. In accidents involving cars or other larger vehicles, 40% are between 18 and 29 years old, 20% are between 30 and 49 years old, and 40% are over 50 years old. It is also worth noting that a third (67%) of pedestrian accidents occurred in patients between 30 and 49 years old.

The type of trauma was classified as blunt or penetrating. The latter happened in 46% of cases, and the most prevalent mechanisms were SW (54.3%) and GSW (67%). Blunt trauma, which was more prevalent in this study, representing 54% of cases, had car accidents (61.1%) and falls from high level (24%) as the main mechanisms. There was no statistically significant difference between the groups.

Among thoracic injuries, the most common was fracture of the costal arches (49%), without the presence of unstable chest, followed by hemothorax (48%), pneumothorax (42%), muscle injury (30%), hemopneumothorax (27% ), pulmonary contusion (14%), diaphragmatic injury (2%), and open pneumothorax (1%). In 2% of the victims, there were only superficial abrasions, 30% had only one thoracic injury, 31% two, 27% three, and 10% more than three thoracic injuries resulting from the trauma.

As for the procedures performed on patients suffering from TT, 31% required only clinical observation, with conservative therapy, analgesia, and ventilatory support, 59% required tube thoracostomy with underwater seal (TTUS), which was the resolving strategy in most cases, and 10% of patients underwent a surgical procedure to open the chest wall (thoracotomy). Furthermore, all patients undergoing thoracotomy remained with a chest tube postoperatively.

TTUS was performed on all victims with hemothorax (n=48) and 16.7% of these required thoracotomies. In pneumothorax, 95.2% (n=40) underwent TTUS and only 11.9% thoracotomy. Duplicity of the two lesions, hemopneumothorax, occurred in 27 patients. The injuries with the lowest percentage of indications for invasive procedures were pulmonary contusion, thoraco-abdominal transition zone (TATZ) injuries, costal arch fractures, and muscle injuries, respectively (Tables 6 and 7). 

**Table 6:** Distribution of Diagnoses according to sex, type of trauma, and procedure.

	Sex	Type of trauma		Procedure Performed		Procedimento Realizado	
	Prevalence	Men	Women	Blunt	Penetrating	Thoracostomy	Thoracotomy
	n (%)	n (%)	n (%)	n (%)	n (%)	n (%)	n (%)
Rib Fracture	49 (49%)	41 (83.7%)	8 (16.3%)	43 (87.8%)	6 (12.2%)	25 (51.0%)	3 (6.1%)
Hemothorax	48 (48%)	43 (89.6%)	5 (10.4%)	13 (27.1%)	35 (72.9%)	48 (100.0%)	8 (16.7%)
Pneumothorax	42 (42%)	37 (88.1%)	5 (11.9%)	18 (42.9%)	24 (57.1%)	40 (95.2%)	5 (11.9%)
Muscle injury	30 (30%)	27 (90.0%)	3 (10.0%)	20 (66.7%)	10 (33.3%)	16 (53.3%)	4 (13.3%)
Hemopneumothorax	27 (27%)	23 (85.2%)	4 (14.8%)	7 (25.9%)	20 (74.1%)	27 (100.0%)	4 (14.8%)
Lung Contusion	14 (14%)	11 (78.6%)	3 (21.4%)	11 (78.6%)	3 (21.4%)	6 (42.9%)	2 (14.3%)
Diaphragm Injury	6 (6%)	6 (100.0%)	0 (0.0%)	1 (16.7%)	5 (83.3%)	3 (50.0%)	2 (33.3%)
Pleural effusion	2 (2%)	1 (50.0%)	1 (50.0%)	2 (100.0%)	0 (0.0%)	2 (100.0%)	0 (0.0%)
Open Pneumothorax	1 (1%)	1 (100.0%)	0 (0.0%)	0 (0.0%)	1 (100.0%)	1 (100.0%)	1 (100.0%)

n: absolute frequency. Source: Authors.

**Table 7:** Distribution of Diagnoses regarding trauma mechanism.

	Assault	Sport	Vehicles	Fall from Level	SW	GSW	Other
Rib Fracture	2 (28.6%)	1 (33.3%)	29 (39.7%)	9 (37.5%)	0 (0.0%)	6 (13.6%)	2 (33.3%)
Hemothorax	1 (14.3%)	0 (0.0%)	7 (9.6%)	3 (12.5%)	22 (35.5%)	14 (31.8%)	1 (16.7%)
Pneumothorax	2 (28.6%)	1 (33.3%)	12 (16.4%)	2 (8.3%)	17 (27.4%)	8 (18.2%)	0 (0.0%)
Muscle injury	1 (14.3%)	0 (0.0%)	13 (17.8%)	5 (20.8%)	7 (11.3%)	3 (6.8%)	1 (16.7%)
Hemopneumothorax	1 (14.3%)	0 (0.0%)	4 (5.5%)	1 (4.2%)	14 (22.6%)	7 (15.9%)	0 (0.0%)
Lung Contusion	0 (0.0%)	1 (33.3%)	5 (6.8%)	4 (16.7%)	0 (0.0%)	3 (6.8%)	1 (16.7%)
Diaphragm Injury	0 (0.0%)	0 (0.0%)	1 (1.4%)	0 (0.0%)	2 (3.2%)	3 (6.8%)	0 (0.0%)
Pleural effusion	0 (0.0%)	0 (0.0%)	1 (1.4%)	0 (0.0%)	0 (0.0%)	0 (0.0%)	1 (16.7%)
Open Pneumothorax	0 (0.0%)	0 (0.0%)	1 (1.4%)	0 (0.0%)	0 (0.0%)	0 (0.0%)	0 (0.0%)

n: absolute frequency. GSW: Gunshot wounds. SW: Stabbing wounds. Source: Authors.

## DISCUSSION

Chest injuries can be superficial, mild, with only the presence of abrasions, or cause immediate risk to the victims, affecting important organs^4^. According to the Advanced Trauma Life Support (ATLS), there are life-threatening injuries that must be promptly treated at the time of their identification in the primary evaluation, such as airway obstruction, injury to the tracheobronchial tree, tension pneumothorax, open pneumothorax, massive hemothorax, and cardiac tamponade. There are also injuries that are potentially fatal, identified during a more thorough evaluation or with the aid of imaging exams, during the secondary evaluation, like simple pneumothorax, hemothorax, flail chest, pulmonary contusion, blunt heart injury, traumatic aortic rupture, injury to the diaphragm, and esophageal rupture^3^.

The majority of thoracic injuries (80%) are represented by pneumothorax, hemothorax and hemo-pneumothorax, which are resolved, in most cases, by TTUS, associated or not with analgesia and ventilatory therapy. There are few cases (10% to 30%) that require more complex procedures, such as thoracotomy, especially when there is massive hemothorax, hemopericardium (cardiac tamponade), hemoptysis, mediastinal widening, respiratory failure due to intense air leakage through the drain, chest pain, and marked hypotension^3^ ^,^ ^4^ ^,^ ^6^ ^,^ ^11^ ^,^ ^12^.

Knowing the trauma and improving its management can improve its prognosis. Studies^13^ ^,^ ^14^ have demonstrated a drop in the fatality rate in the First World War (24.6%), when compared to the Second (12%) and the Vietnam (5%) Wars, being attributed to the knowledge and management of the main chest injuries.

We found that TT was more prevalent in young men of economically active age. We observed data similar to the literature regarding gender, where Aline et al. (2023) presented an incidence of 82.2% of men, Broska Júnior et al. (2017) of 84.7%, Jorge Carlos et al. (2022) of 86.7%, and Narayanan et al. (2018), with M:F ratio of 8:1^12^ ^,^ ^13^ ^,^ ^15^ ^,^ ^16^. Likewise, the average age of 38.3 years was similar to the work of Zanette et al. (2019), of 39.98 years, Broska Júnior et al. (2017), of 34.7 years, and Narayanan et al. (2018), 37.82 years^6^ ^,^ ^12^ ^,^ ^16^.

Regarding the type of trauma, traffic accidents, especially with motorcycles, were the main mechanism of TT, respectively 33% and 22%, which is in agreement with the literature^6^ ^,^ ^12^ ^,^ ^13^ ^,^ ^16^. Traffic accidents currently represent a serious public health problem, especially in Western countries, where such events constitute 80-85% of the TT fundamental causes^13^. According to Zanette et al. (2019), higher rates of traffic accidents are due to the association of external factors, such as speeding and alcohol abuse; and Santos et al. (2008), on their turn, link them to aspects such as vehicle conditions and improper use of safety equipment^6^ ^,^ ^14^. 

We observed the lack of updated epidemiological data in the literature, despite the geographic profile of patients sustaining TT in Sergipe. HUSE is located in Aracaju, capital of Sergipe, and is the main reference for trauma patients in adults, especially for more complex injuries. In the present study, most patients came from Aracaju and adjacent regions of the state, such as East Sergipe, accounting for approximately 56% of the visits. We also noted a higher prevalence of patients from the Central-South region of the State (20%). Such patients required longer travel time for care, which may have negatively influenced their prognosis.

As mentioned, in our study, blunt (closed) trauma was the most prevalent, but there was no statistically significant difference between the traumas. Other studies^6^ ^,^ ^13^ ^,^ ^17^ ^,^ ^16^ have found statistically significant differences, pointing to blunt trauma as the most prevalent, with percentages ranging from 71.2% to 91%.

In cases of penetrating (open) TT, we observed a greater number of injuries from sharp weapons, mainly from blades (knives), reaching 96% of these cases, and the most common thoracic injuries from these injuries were hemothorax (72.9%) and pneumothorax (51.7%), agreeing with the literature^3^ ^,^ ^6^ ^,^ ^12^, which associates such diagnoses mainly with open trauma. 

On the other hand, in the closed TT, automobile accidents (61.1%) and falling from a high level (24%) were the most prevalent and the most common associated injury was rib fracture, present in 39.7% and 37.5%, respectively. Muscle injuries appeared in 1/5 of these traumas. Both Zanette et al. (2019) and Broska Júnior et al. (2017) identify falls as the second most prevalent mechanism in blunt trauma, being an important cause in old age^6^ ^,^ ^12^. In our study, 62% of patients who fell from a level were over 50 years old, with a statistically significant correlation.

Most patients were treated without the need for an open surgical approach, in accordance with the literature^6^ ^,^ ^12^ ^,^ ^13^ ^,^ ^17^ ^,^ ^18^. Only 10% underwent thoracotomy due to massive hemothorax or hemopneumothorax. According to ATLS, less than 10% of patients with closed TT require surgical treatment and 15% to 30% of penetrating injuries will require surgery^3^ ^,^ ^13^. 

Many authors recommend thoracotomy in all patients with TT and hemodynamic instability, in addition to patients with hemothorax classified as medium, considering it the best way to perform intrathoracic hemostasis and prevent future complications^13^ ^,^ ^18^ ^,^ ^19^.

TTUS was the most frequently performed intervention in our patients to resolve intrathoracic injuries and complications, being the basis of TT treatment. Furthermore, the conservative approach, without the need for thoracotomy intervention or TTUS, was present in 31% of patients, where monitoring, oxygen therapy, and analgesia were carried out according to each case, without increasing mortality or worsening prognosis. Even though it is a simple procedure, TTUS can lead to a considerable number of complications, especially when performed by non-specialists^18^.

## FINAL CONSIDERATIONS

TT is a highly challenging pathology due to the complexity of the resulting injuries and because most of them are preventable causes of death. In the present study, car accidents were the most common kinematics of trauma, especially in healthy men of economically active age. Urban violence was also an important indicator, representing 47% of all events. Performing TTUS was decisive in many of these cases and should be promptly instituted when necessary.

Many of the correlations found could not be statistically significantly proven. One of the explanations for this can be given by the limitation of the sample. Another limitation of our study was the lack of pediatric patients. These were often referred to other specialized services in the capital aimed at this population. Furthermore, critical cases that died within a short period of time after admission were not included in the study for ethical reasons, as it was not possible to interview patients or family members.

In this sense, we recommend the continuation of more studies that overcome these limitations, and we hoped that this study can help in the organization of hospital protocols and the implementation of public preventive and awareness policies, in addition to improvements in the diagnosis and management of the main thoracic injuries.

**LEGENDS**
ACSCOT: American College of Surgeons Committee on Trauma
ATLS: Advanced Training Life Support
SW: Stabbing wound
GSW: Gunshot Wound
HUSE: Sergipe Emergency Hospital / João Alves Filho Hospital
IAF: Informed Assent Form
ICF: Informed Consent Form
TTUS: Tube Thoracostomy with Underwater Seal
TT: Thoracic Trauma
TATZ: Thoraco-Abdominal Transition Zone
